# An Analysis of Demographic and Triage Assessment Findings in Bushfire-Affected Koalas (*Phascolarctos cinereus*) on Kangaroo Island, South Australia, 2019–2020

**DOI:** 10.3390/ani11113237

**Published:** 2021-11-12

**Authors:** Evie Dunstan, Oliver Funnell, Jenny McLelland, Felicity Stoeckeler, Elisa Nishimoto, Dana Mitchell, Sam Mitchell, David J. McLelland, Jerome Kalvas, Lynley Johnson, Claire Moore, Lauren J. M. Eyre, Amanda McLune, Ian Hough, Ludovica Valenza, Wayne S. J. Boardman, Ian Smith, Natasha Speight

**Affiliations:** 1 School of Animal and Veterinary Sciences, Faculty of Sciences, Roseworthy Campus, University of Adelaide, Adelaide, SA 5371, Australia; evie.dunstan@adelaide.edu.au (E.D.); jmclelland@zoossa.com.au (J.M.); dmclelland@zoossa.com.au (D.J.M.); jkalvas@zoossa.com.au (J.K.); wayne.boardman@adelaide.edu.au (W.S.J.B.); ismith@zoossa.com.au (I.S.); 2 Zoos South Australia, Frome Rd, Adelaide, SA 5001, Australia; ofunnell@zoossa.com.au (O.F.); ljohnson@zoossa.com.au (L.J.); 3 Kangaroo Island Veterinary Clinic, Kingscote, SA 5223, Australia; felicity.stoeckeler@gmail.com (F.S.); elisa@kangarooislandvet.com (E.N.); 4 Kangaroo Island Wildlife Park, Parndana, SA 5220, Australia; danamitchell@kangarooislandwildlifepark.com (D.M.); sammitchell@kangarooislandwildlifepark.com (S.M.); 5 South Australian Veterinary Emergency Management, Bridgewater, SA 5155, Australia; claire.moore@flinders.edu.au; 6 Royal Society for the Protection of Cruelty to Animals, South Australia (RSPCA SA), Lonsdale, SA 5160, Australia; leyre@rspcasa.org.au; 7 Australian Defence Force, Army Headquarters, Canberra, ACT 2600, Australia; Admin@vetmacs.com.au; 8 Cleland Wildlife Park, National Parks and Wildlife Service South Australia, Department for Environment and Water, South Australian Government, Crafers, SA 5152, Australia; Ian.Hough@sa.gov.au; 9 Australia Zoo Wildlife Hospital, Beerwah, QLD 4519, Australia; ludov@wildlifewarriors.org.au

**Keywords:** burn, mortality, Phascolarctidae, rescue, trauma, wildfire

## Abstract

**Simple Summary:**

In the 2019–2020 Australian bushfires, Kangaroo Island, South Australia, experienced catastrophic bushfires that burnt approximately half the island, with an estimated 80% of the koala population lost. During and after the fires, koalas presented to a designated triage facility over a span of 10 weeks, with 50.2% during the first 14 days of the bushfire response (304 records available). Burns were observed in 67.4% of koalas, with the majority (60.9%) classified as superficial burns, primarily affecting the limbs and face. Poor body condition was recorded in 74.6% of burnt koalas and dehydration in 77.1%. Negative final outcomes (death or euthanasia, either at triage or at a later date) occurred in 45.6% of koalas and were significantly associated with higher mean burn score, maximum burn severity, number of body regions burnt, poor body condition score, and dehydration severity. The findings of this retrospective study may assist clinicians in the field with decision making when triaging koalas in future fire rescue efforts.

**Abstract:**

In the 2019–2020 Australian bushfires, Kangaroo Island, South Australia, experienced catastrophic bushfires that burnt approximately half the island, with an estimated 80% of the koala population lost. During and after the event, rescued koalas were triaged at a designated facility and a range of initial data were recorded including rescue location and date, sex, estimation of age, body condition and hydration, and assessment of burn severity (*n* = 304 records available). Koalas were presented to the triage facility over a span of 10 weeks, with 50.2% during the first 14 days of the bushfire response, the majority of which were rescued from regions of lower fire severity. Burns were observed in 67.4% of koalas, with the majority (60.9%) classified as superficial burns, primarily affecting the limbs and face. Poor body condition was recorded in 74.6% of burnt koalas and dehydration in 77.1%. Negative final outcomes (death or euthanasia, at triage or at a later date) occurred in 45.6% of koalas and were significantly associated with higher mean burn score, maximum burn severity, number of body regions burnt, poor body condition score, and dehydration severity. The findings of this retrospective study may assist clinicians in the field with decision making when triaging koalas in future fire rescue efforts.

## 1. Introduction

In the 2019–2020 Australian bushfires, often referred to as the Black Summer fires, millions of hectares of land were burnt across Australia [1]. The Worldwide Fund for Nature estimated that Australia-wide, 30 billion animals were impacted, including more than 60,000 koalas (*Phascolarctos cinereus*) [2]. In South Australia, the largest bushfire occurred on Kangaroo Island (KI) (4405 km^2^ in area), with approximately 50% of the island (2110 km^2^) burnt [3]. Of this, 85% was native vegetation inhabited by koalas and as a result, up to 80% of the estimated 50,000 koalas on KI perished, with a population of approximately 5000–10,000 individuals remaining [4].

The koala population on KI originated from 18 individuals introduced from French Island, Victoria, in the early 1920s [5,6]. Whilst the population has been regarded as overabundant since 1965 [7,8], their recently confirmed status as free of *Chlamydia pecorum* infection and disease [9] distinguishes KI koalas from other mainland populations [10]. Koalas are distributed across the island, particularly in areas in which manna gum (*Eucalyptus viminalis*) is present; however, they have also been observed in the commercial blue gum (*Eucalyptus globulus*) plantations [4,8].

As the recent bushfire progressed on KI, a large coordinated state emergency response was mounted, similar to that described for the bushfires in Victoria, Australia [11], and an important part of this response was the rescue and triage of wildlife, including koalas. In the 2019–2020 Victorian fires, outcomes for rescued koalas included release of 42% of koalas within 24 h of triage, and euthanasia of 20% of koalas at triage [11]. However, reports of the specific clinical findings of koalas at triage following their rescue from bushfires are limited. Smoke inhalation and burns were identified in koalas rescued from a 1994 New South Wales bushfire [12], with the burns particularly affecting the footpads [12]. Footpad burns in koalas have also been recently reported in a South Australian study of various pathological findings in necropsied koalas [13], and presumably occur due to contact with burnt tree trunks and when they descend to the ground. Stress as a result of bushfires has also been reported in affected koalas, with increased faecal cortisol metabolites detected in samples from burnt koalas [14]. In other Australian wildlife, mortality due to shock, starvation, and predation has been identified in woylie (*Bettongia penicillata*) and tammar wallabies (*Macropus eugenii*) [15]. Longer term impacts of bushfires on wildlife populations such as habitat loss and decreased feed availability due to destruction of trees and other vegetation are also major concerns that have been investigated [16]. Whilst successful rehabilitation and release of koalas post-fire have been reported, showing the potential efficacy of bushfire rescue efforts [11,12,17], high mortality of wildlife is inevitable in high-severity bushfires [4,11].

This retrospective study aimed to describe the timeline and location of Kangaroo Island koalas rescued in the 2019–2020 bushfires, the prevalence, severity and extent of burns recorded at triage, risk factors such as sex and age, and prognostic indicators associated with final outcomes.

## 2. Materials and Methods

### 2.1. Koala Rescue Data

During and after the 2019–2020 KI bushfires, koalas and other wildlife in fire-affected regions were rescued by members of the public, emergency personnel, and dedicated wildlife rescue groups. Wildlife were taken to a designated triage and treatment centre established at the Kangaroo Island Wildlife Park (KIWP) in Parndana, KI. On arrival, koalas received an animal identification number (*n* = 304 records available), and the rescue date and location were recorded. Rescue dates of koalas (*n* = 303) were used to create a timeline in relation to key events within the fire period. Rescue locations (*n* = 89) were mapped using Google Earth Pro v7.3.4 software (Google LLC, Mountain View, CA, United States) either as exact road addresses and intersections (*n* = 12), or as the centre of roads (*n* = 43) and approximate areas (*n* = 34). Images of the fire severity and extent, and vegetation type, developed by the Department for Environment and Water [4], were overlaid onto the maps.

### 2.2. Triage Data

The clinical examination record for each koala was completed by veterinarians and included sex, age, body condition score (BCS), hydration status, descriptions of burns or other injuries, and outcomes. To allow for statistical analysis of clinical data, standardised scoring systems were developed retrospectively for age, body condition, hydration status, and burns descriptions to account for a wide range of descriptive terms used by triage staff.

Ages of koalas were categorised to account for the various ageing methods used by triage staff. These included koala life stage (independent juvenile, young adult and adult), stating an estimated age in years based on a tooth wear ageing guide [18], or recording the koala tooth wear class (TWC) according to the system of Martin and Handasyde (1999) [6] (Table 1).

Body condition of koalas was standardised as poor (<3) or adequate (≥3) from various scales used to record BCS at triage. These included domestic animal scales which score body condition 1–5 or 1–9, whereby any score less than the median is underweight and above is overweight, as well as a specific koala scale 1–5, whereby 5 is excellent, and < 3 is poor or emaciated [18]. In many cases, the scoring method used was not stated, hence there was a necessity to categorise body condition only as poor or adequate for this study.

Hydration status was scored 0–3 to account for various ways that hydration had been recorded at triage, including various descriptive terms or a percentage dehydration assessed by methods presented in Blanshard and Bodley (2008) [18] (Table 2).

From triage records, koalas were grouped into those with and without burns reported. A wide range of terms were used to describe burn severity and are presented in Table 3. These terms were used to classify burns on a scale of 1–3 (superficial, partial thickness, and full thickness) based on the system used in dogs and cats [19], and adapted from that used in humans [20]. In some cases, burn severity was unable to be classified as no further details of burns were provided, or non-specific terms such as ‘healed’ or ‘healing’ were used. Burn severity was assessed over five body regions: right forelimb, left forelimb, right hindlimb, left hindlimb, and head/face; burns to the trunk were only recorded in four koalas. Following this, three measures of burn severity were calculated: mean burn score across the five body regions (0.2–3), maximum burn severity of the five body regions (1–3), and number of regions burnt (1–5).

Outcomes of koalas (*n* = 239) were recorded on triage records or final patient records. For this study, these were categorised as either positive or negative outcomes, where positive included release, soft release, and retained in captivity, and negative included death or euthanasia.

### 2.3. Statistical Analysis

Descriptive statistics were generated for data using IBM^®^ SPSS^®^ 27 (IBM, Armonk, NY, USA) and Microsoft Excel^®^ (Microsoft, Redmond, WA, USA). Continuous data were assessed for normality, and outliers were identified and excluded using Dixon’s test. If continuous data were normally distributed, independent samples t-test for means comparison were used. If not normally distributed, then non-parametric Mann–Whitney U means comparison was used. For associations where both variables were categorical, Pearson’s Chi-square tests for association and Odds ratios were undertaken. Spearman’s correlation tests were utilised to identify relationships between ordinal and continuous variables. Results were considered significant at *p* < 0.05.

## 3. Results

### 3.1. Koala Rescue Timeline and Locations on Kangaroo Island

The Kangaroo Island bushfire started as two separate fires in the Menzies region on 7 December 2019 and in Duncan region on 21 December 2019, with a third fire later igniting in Ravine des Casoars on 30 December. Following this, the fires spread from the northern and western districts towards the centre of the island, forming one large fire, which then moved in an easterly direction in the later stages [21]. Fire progression ceased on 13 January, followed by declaration of all fires as contained, and safe, on 21 January and 6 February, respectively.

**Figure 1:**
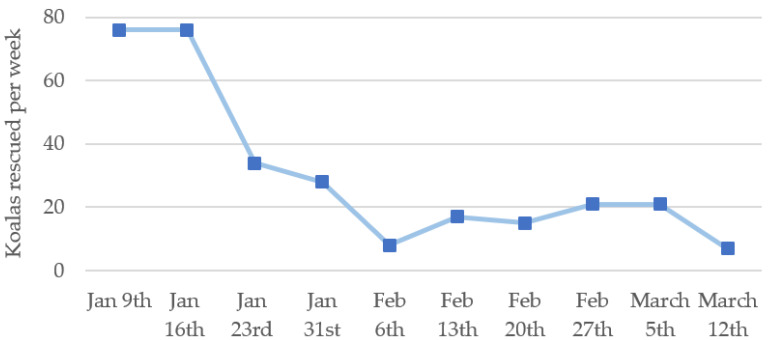
Number of recorded koala rescues per week. First recorded koala rescue in this study occurred on 9 January 2020. Fire progression ceased on 13 January, followed by declaration of all fires as contained, and safe, on 21 January and 6 February, respectively.

A total of 304 complete triage records, spanning from 9 January to 15 March (67 days inclusive), were available from Kangaroo Island koalas rescued from the bushfires. This was from over 600 koalas rescued from 1 January, the remaining having incomplete or missing triage records. For the 304 koalas, between day 1 (9 January) and the ceasing of the fire progression on day 5 (13 January), 20.5% of koalas presented for triage (initial clinical examination), and 50.2% by day 14 (22 January) (Figure 1). The highest daily number of triaged koalas (*n* = 18) was recorded on day 12 (20 January), the day before the fires were declared contained. Koalas continued to present for triage until 15 March, in lower numbers following the fires being declared safe on day 29 (6 February). Overall, 67.4% (205 of 304) of rescued koalas were found at triage examination to have sustained burns from the bushfires.

**Figure 2:**
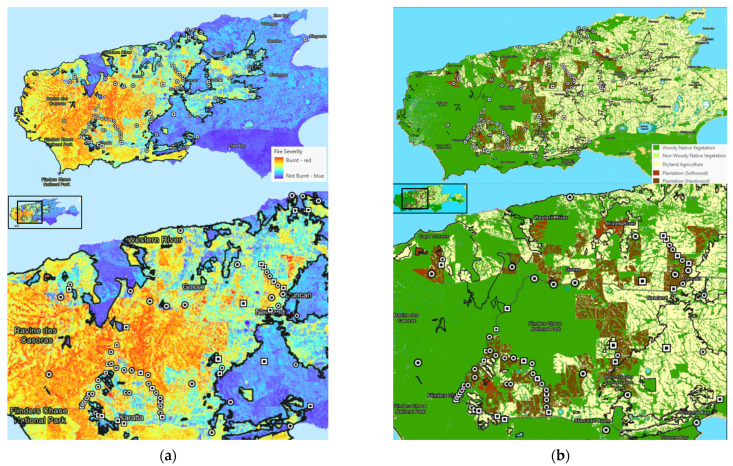
Map of Kangaroo Island created using Google Earth Pro v7.3.4 software to show recorded locations of koalas with burns (circle) and without burns (square) rescued from the 2019–2020 bushfires, and overlaid with adapted images developed by Department for Environment and Water, Government of South Australia [4] from base maps at data.sa.gov.au under Creative Commons Attribution 4.0 licence, demonstrating: (**a**) fire ground and fire severity, and (**b**) fire ground and vegetation type. Box in inset refers to area shown in enlarged (lower) maps.

Based on fire severity, the rescue locations (*n* = 89) recorded for many koalas were in close proximity to low-severity fireground, patchy fireground, or edges of fireground (Figure 2a), with clusters around Duncan and Karatta, in close proximity to a high number of eucalypt (hardwood) plantations (Figure 2b). Very few koala rescue locations recorded were within high severity fireground locations in the far western end of the island, which primarily consisted of woody native vegetation. From the available data, there was no apparent relationship between the presence of burns in triaged koalas and their rescue location in relation to fireground, fire severity, or vegetation type.

### 3.2. Clinical Examination Findings at Triage

Of koalas with sex recorded at triage (*n* = 300), 142 (47%) were female and 158 (53%) were male. Of koalas with age estimated, 49 (20.2%) were independent juveniles, 93 (38.3%) were young adults and 101 (40.6%) were adults (*n* = 243). No statistical difference was found for the total number of female and male koalas rescued (*p* = 0.356) or for each age category (*p* = 0.346).

**Figure 3:**
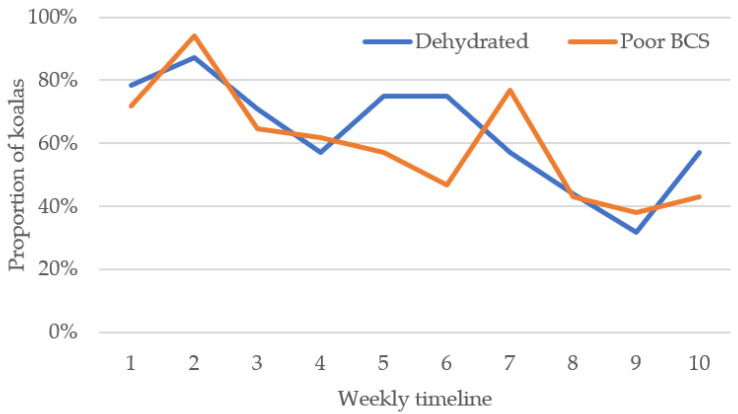
Weekly proportions of koalas clinically assessed with poor body condition (BCS) and dehydration at the time of triage.

Of koalas with BCS recorded at triage (*n* = 263), 181 (68.8%) were considered to have a poor body condition. There was no association of sex *(p =* 0.147) or age (*p* = 0.355) with body condition. There was a moderate negative correlation for the proportion of koalas with poor body condition over the weeks of the bushfire response, whereby body condition of koalas presenting at triage improved over time (r = −0.772, *p* = 0.009) (Figure 3).

Where hydration status of koalas had been estimated, 159 of 226 (70.4%) were assessed as dehydrated (score ≥ 1), with a mean score for dehydrated koalas of 1.88 (±0.05) out of 3. Mild, moderate and severe dehydration was recorded in 28.3% (*n* = 45), 55.4% (*n* = 88) and 16.4% (*n* = 26) of koalas, respectively. There was no association of hydration status with sex *(p =* 0.707) or age (*p* = 0.433). The proportion of dehydrated koalas was negatively correlated with time (weeks), whereby fewer koalas were dehydrated as triage continued (r = −0.788, *p* = 0.007) (Figure 3). However, the severity of dehydration in koalas stayed constant over the duration of the bushfire response (r = −0.042, *p* = 0.599).

**Figure 4:**
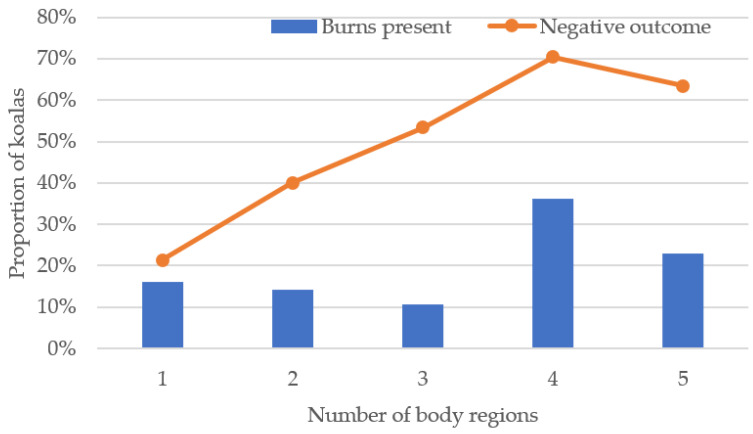
Proportion of koalas with burns to one, two, three, four and five regions and those with negative outcomes.

Mean burn score (*n* = 128) calculated for koalas across the five body regions (range 0.2–3) was 0.65 (±0.05). Mean maximum burn severity (*n* = 128) was 1.5 (±0.06), and superficial, partial thickness, and full thickness burns were recorded in 60.9%, 28.1%, and 10.9% of koalas, respectively. The mean number of regions burnt for koalas in which this was recorded (*n* = 205) was 3.4 (±0.1) out of 5, with burns to four regions most common (Figure 4). The limbs were the most common body regions burnt. Burns to each of the limbs occurred at a prevalence of 71.2% (left forelimb), 68.3% (right forelimb), 72.2% (left hindlimb), and 77.6% (right hindlimb). Included with the limb burns were specific reports of damaged, loose, or lost claws and nailbed damage in 25 koalas, and burns to the hands and feet in three koalas. The head/face region was significantly less affected than the limbs (*p* = 0.02), whereby 46.8% of burnt koalas sustained burns to the head/face, mainly around the ears, eyes, nose, lips and chin. Only four individuals were reported with burns to the body, which included areas such as the rump, groin, flank, abdomen, and scrotum.

**Figure 5:**
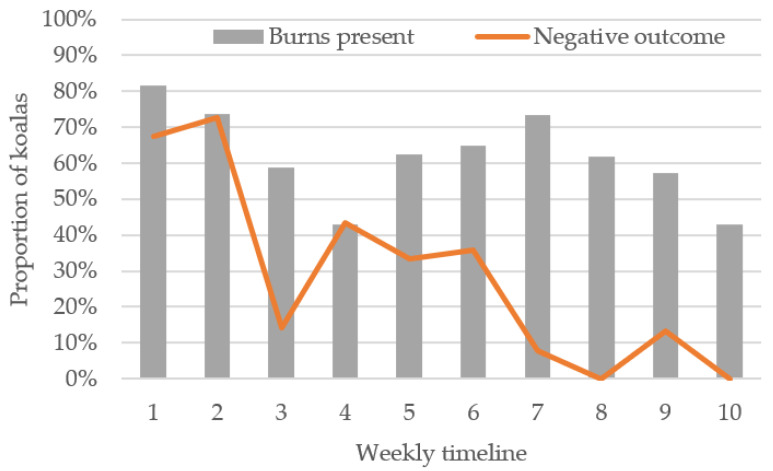
Weekly proportion of koalas with burns as triage progressed, and weekly proportion of koalas with negative outcomes.

The weekly proportion of koalas with burns remained relatively constant over the duration of the bushfire response (r = −0.565, *p* = 0.09), although the highest weekly proportion of koalas with burns was in the first week (81.6%), and the lowest in the fourth week (42.9%). A secondary peak of a high proportion of koalas presenting with burns (73.3%) occurred in the seventh week (Figure 5).

**Figure 6:**
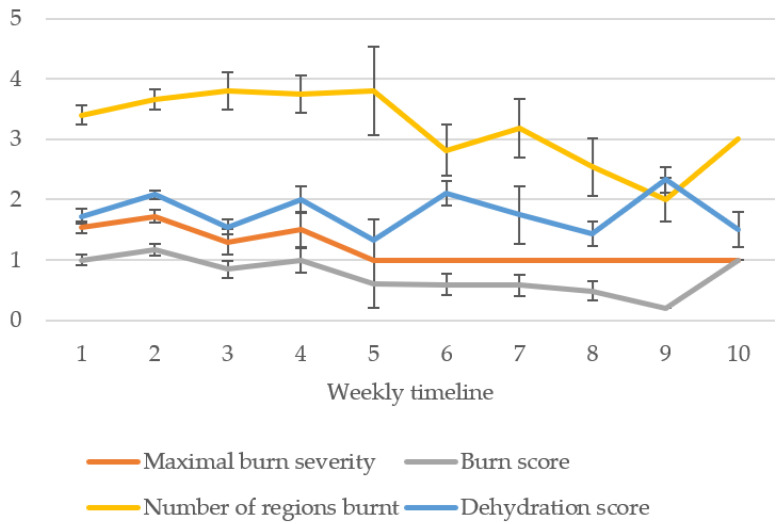
Weekly mean burn score (0.2–3), mean maximum burn severity (1–3), mean number of regions burnt (1–5), and mean dehydration score (1–3), for koalas assessed at triage (standard error bars shown).

However, weekly mean burn score decreased as triage progressed, showing negative correlation (r = −0.266, *p* = 0.002, *n* = 128) (Figure 6). Weekly mean maximal burn severity and weekly mean number of regions burnt also decreased over time, both demonstrating negative correlations (r = −0.256, *p =* 0.004, *n* = 128 and r = −0.148, *p =* 0.034, *n* = 205, respectively). No significant association was found between burns status and sex *(p =* 0.732) or age *(p =* 0.455) of koalas. However, a significant association was found between burns status and body condition, whereby a higher proportion of koalas with poor body condition presented with burns (74.6%) compared to koalas with adequate body condition (48.5%) (*p* = 0.009), with burnt koalas 2.1 times more likely to have poor body condition. A significant association was also found between burns status and hydration status, whereby a higher proportion of burnt koalas were assessed as being dehydrated (77.1%) compared with unburnt koalas (55.6%) (*p =* 0.001), with burnt koalas 2.7 times more likely to be assessed as dehydrated.

### 3.3. Outcomes of Triaged Koalas and Prognostic Factors

The final outcome of koalas following triage, and for some individuals after treatment whilst in care, was recorded for 239 animals. Negative outcomes were recorded for 109 (45.6%) koalas, including triage euthanasia (7.1%), triage death (1.3%), dead on arrival (0.4%), euthanasia at a later date (16.7%), or death at a later date (20.1%). Positive outcomes were recorded for 130 (54.4%) koalas, including release at a later date (44.8%), soft release (9.2%) or retained in captivity (0.4%). There was a strong negative correlation between proportion of negative outcomes and weeks since rescues began, whereby more koalas had positive outcomes later in the bushfire response (r = −0.857, *p* = 0.002, *n* = 238) (Figure 5).

A significant association between burns status and outcome of koalas was found (*p* < 0.001), whereby a higher proportion of burnt koalas had negative outcomes (54.4%) compared with koalas without burns (28.4%), with burnt koalas three times more likely to have negative outcomes. Koalas with negative outcomes had significantly higher mean burn score, maximal burn severity, and number of regions burnt (Table 4). The proportion of negative outcomes for maximum burn severity categories superficial, partial thickness, and full thickness burns were 38.2%, 96.7% and 90%, respectively (*n* = 108). Additionally, a strong positive correlation was found between proportion of koalas with negative outcomes and number of regions burnt (r = 0.9, *p*-value = 0.037, *n* = 158), with negative outcomes affecting a high proportion of koalas with four regions burnt (Figure 4). Body condition was significantly associated with outcome, whereby a higher proportion of koalas with adequate body condition had positive outcomes (73%) compared with koalas with poor body condition (49%) (*p* < 0.001). Hydration status was also important, whereby koalas with negative outcomes had a significantly higher dehydration score (Table 4), and a significant strong positive correlation was found between dehydration severity and proportion of koalas with negative outcomes (r = 1, *p* < 0.001, *n* = 130). There was no significant association between outcome and sex (*p* = 0.477) or age (*p* = 0.541) of koalas.

## 4. Discussion

This retrospective study analysed the demographic and clinical data of Kangaroo Island koalas that presented for triage assessment during the 2019–2020 bushfires, finding that more than half of the 304 koalas in the study cohort presented within the first two weeks of the bushfire response. Day 12 was the peak of recorded koala triages, which is similar to a previous study in which the highest number of koalas were rescued on day 17 after a bushfire in Victoria in 2007 [22]. This suggests that wildlife rescue efforts should begin as soon as a fireground is safe to access by trained and experienced personnel, and focus on the initial few weeks. However, it must be recognised that during this initial period, there were also the highest proportions of koalas with negative outcomes. Parrott et al. (2021) likewise reported a high number of wildlife euthanised at triage (20%) during the Victorian bushfires [11]. Despite this, humane euthanasia by veterinarians is preferable compared with animals dying, from a welfare perspective.

The locations of wildlife rescued from bushfires in relation to fireground, fire severity, and vegetation type have not previously been described. Flinders Chase National Park (FCNP) in the western region of Kangaroo Island was burnt at high severity [4] and, prior to the KI bushfires, had been identified as having a high density of koalas [23]. Despite this, few koala rescues occurred from FCNP and other high fire severity areas, likely due to the associated danger for emergency and rescue personnel to access, and that koalas are less likely to survive higher severity fires. A report by Gill and Catling (2002) found that high severity fires particularly affect canopy dwelling species such as koalas, whereas lower severity fires affect ground dwelling and understorey animal species [24]. The highest number of koala rescues occurred in low fire severity areas of the island, as well as in or near eucalypt plantations. The latter is likely due to their high habitat suitability for koalas, which is supported by the high pre-fire numbers of koalas reported within KI blue gum plantations [25]. Additionally, larger road networks exist around eucalypt plantations compared with native vegetation areas such as FCNP, thereby providing safer access to rescue teams, and likely leading to the higher number of rescues around plantations and lower numbers in FCNP. These observations highlight the necessity to consider many variables such as safety, fire severity, vegetation type, and access when deploying rescue teams. However, it must also be recognised that there were a limited number of koalas in this study cohort with precise rescue locations recorded, and regions such as FCNP may well be underrepresented.

Burns affected 67.4% of koalas and the mean maximum burn severity was 1.5 out of 3, with a score of one corresponding to the superficial layer of the skin (epidermis), and two to a partial thickness burn extending into the dermis [19]. It may be that koalas with burns of lesser severity are more likely to be rescued, either due to the ability to escape to the edge of the fireground, or that they were dwelling in areas that experienced lower fire severity. Likewise, the underrepresentation of high severity burns that presented at the triage facility may be due to the likelihood that koalas with higher severity burns died in regions of the island that were inaccessible to personnel due to fire severity and/or terrain, or before being able to be reached by rescue teams. The reality of any bushfire response is that only a small proportion of affected wildlife (approximately 1% of the KI koalas that perished) are able to be rescued due to the inherent dangers of bushfire for emergency personnel.

Burns to the limbs of koalas were found to be common, with many koalas having multiple limbs affected, and the number of body regions burnt correlated with increasing proportions of koalas with negative outcomes. Although only documented in a few cases, burns to the limbs were most often characterised by palmar and plantar burns on the footpads extending to damage of the claws, rather than burns to the proximal furred regions of the limbs (O Funnell, J McLelland, author obs.). This is similar to previous reports of footpad burns in bushfire rescued koalas in New South Wales [12], and the Mount Lofty Ranges, South Australia [13]. These footpad and claw burns are likely to be particularly associated with poor prognosis in koalas, rendering them unable to climb trees and access feed. This is comparable to a study of sheep affected by bushfire that suggested worse prognoses for burns to the hooves [26], as they decrease the animal’s ability to access feed and water. However, burns to the head and face were also found to affect almost half of koalas and may have equally poor prognosis, particularly where the mouth and eyes are involved.

Adult koalas accounted for the majority of koalas presented at triage, and no significant difference in male and female koala rescues were found. Wallis (2013) reported similar findings from the rescue of 147 koalas from a bushfire in Victoria in 2007 [22], where the majority of rescued koalas were aged between three and five years old with sex proportions being close to equal. The high proportion of koalas assessed as having poor body condition at triage was unexpected when considering findings from a study that assessed the body condition of KI koalas between 2014 and 2017, and found that 84.1% of koalas were in excellent body condition [27]. However, the recent reports of the overabundance of koalas on KI [8,23,25], suggest that decreasing feed availability prior to the bushfires could explain the high prevalence of poor body condition. Yet, due to the variability of body condition recording methods at triage, some level of error likely exists within the proportion of koalas with poor body condition found in this study.

A significantly higher proportion of koalas assessed as having poor body condition at triage had burns, compared with those having adequate body condition. Whilst this could suggest that koalas with poor body condition were less fit for escape from the fire, perhaps due to underlying disease, KI koalas have previously been found to be relatively disease-free [9,28]. Hence, further investigation into the relationship between body condition and burns is needed to confirm poor body condition as a risk factor. Considering the decreased feed availability that occurs after bushfire [16], the decreasing weekly proportion of koalas with poor body condition presenting at triage as the bushfire response progressed was unexpected; however, this aligned with the decreasing numbers of burnt koalas as well. It may be that koalas that had adequate body condition were more likely to survive through to the later stages of rescue efforts with only minor burns, and that these koalas had moved into areas with unburnt food trees. Overall, poor body condition was strongly associated with negative outcomes of koalas, suggesting that poor body condition could be used in conjunction with other prognostic indicators to assist assessment of koalas at triage.

Dehydration was diagnosed in the majority of koalas assessed at triage, likely due to a combination of exposure to heat created by the bushfires, flight from the fires, and high ambient temperatures. Previous physiological studies in koalas have shown that water loss can be significant in this species as ambient temperature increases [29]. There was a higher proportion of koalas with burns that were also dehydrated compared to koalas without burns, and a significantly higher proportion of severely dehydrated koalas that had negative outcomes, indicating that hydration status is an important prognostic indicator for bushfire-rescued koalas.

Positive outcomes were seen for more than half (54.4%) of rescued koalas in the study cohort, providing reassurance that bushfire rescue efforts can be effective. However, positive outcomes increased over the course of the bushfire response, when burn severity measures, poor body condition, and dehydration were decreasing in koalas presented at triage. Little is published of the outcomes of rescued koalas from previous bushfires; however, a recent study of wildlife rescued from the Black Summer bushfires in Victoria reported that 42% of rescued wildlife were released within 24 h of triage [11]. As release of KI koalas was more likely to occur for individuals triaged later in the bushfire response, it suggests that some koalas can find means to survive bushfires, despite minor burns. However, for many of the KI koalas rescued, predominantly in the early stages of the response, triage records are missing or incomplete, and this could underestimate the number of koalas that died or were euthanised during the bushfire response.

The correlation of increasing mean burn score and maximal burn severity with increasing proportion of negative outcomes indicated that both measures of burns severity are appropriate prognostic factors to consider for koalas. In addition to this, the correlation between the increasing number of regions burnt and negative outcome suggests that the extent of burns should be assessed in conjunction with burn severity when determining an animal’s prognosis. Spielman (1994) proposed that a good prognosis exists when burns are less than 15% of an animal’s body [30]. Development of a specific method for estimation of burn extent in koalas would need to take into account the crucial role of the footpads and claws in the survival of this arboreal species, and would be very useful for informing triage in future bushfire responses.

## 5. Conclusions

The rescue of koalas from the 2019–2020 bushfires in Kangaroo Island, South Australia was a significant undertaking requiring the coordination of emergency personnel, veterinary teams, and wildlife rescue groups, similar to that described in Parrott et al. (2021) in Victoria for the same bushfire season. Despite the inherent challenges of thorough record keeping by a large number of personnel during an emergency response, this study has been able to retrospectively analyse the available demographic and triage data from 304 rescued KI koalas. We found that koalas with more severe burn parameters are more likely to have negative outcomes, highlighting the importance of burn severity and extent as prognostic indicators. Additionally, consideration of dehydration and body condition of koalas may be useful when assessing koalas at triage. Based on the findings of this study, a prognostic algorithm could be developed that would be beneficial for the efficacy of future rescue efforts. This could be based on mean burn score, maximum burn severity, number of regions affected, dehydration severity, and body condition score. Such an algorithm could also incorporate a method to more accurately estimate the extent and importance of burnt areas on the koala body. This would assist veterinarians in determining the prognosis of burnt koalas and likelihood of rehabilitation following hospitalisation. Furthermore, development of a standardised format of rescue and triage data collection, compared with open style triage forms, would create opportunities for further research of the clinical findings of affected koalas and wildlife, and contribute to our preparedness for future bushfires.

## Figures and Tables

**Table 1:** Koala age groups corresponding to life stage, estimated age, and tooth wear class.

Age Group	Life Stage	Age (Years)	Tooth Wear Class [6]
1	Juvenile (independent)	1–2	I
2	Young adult	3–4	II, III
3	Adult	5+	IV, V, VI, VII

**Table 2:** Hydration status of koalas based on descriptions recorded at triage.

Hydration Score	State of Hydration as Recorded at Triage
0	Adequate, fine, normal, good, not clinically dehydrated
1	Mild, slightly, fair, <5%
2	Moderate, dehydrated, D+, poor 5–10%
3	Severe, >10%

**Table 3:** Burn severity classification based on descriptions used in triage records.

Burn Severity	Classification	Terms Used in Triage Records
0	Not burnt	No burns, nil, OK, no abnormalities detected, no significant findings, no entry
1	Superficial burn	Superficial, singed fur, minor, mild, minimal, small, scorched
2	Partial thickness burn	Superficial partial thickness, deep partial thickness, moderate, medium, ulcer, scab
3	Full thickness burn	Full thickness, severe, deep, skin loss, eschar

**Table 4:** Associations of positive (+) and negative (−) outcomes of rescued Kangaroo Island koalas, with burns and dehydration.

Parameter	Outcome of Koalas	Koalas (*n*)	Mean	Standard Error	Significance (*p*-Value)
Mean burn score	+	44	0.55	0.05	*p* < 0.001
(0.2–3)	−	64	1.21	0.75	
Maximum burn severity	+	44	1.07	0.05	*p* < 0.001
(1–3)	−	64	1.73	0.09	
Number of regions burnt	+	72	2.86	0.19	*p* < 0.001
(1–5)	−	86	3.81	0.13	
Hydration score	+	105	0.92	0.09	*p* < 0.001
(0–3)	−	80	1.91	0.11	

## Data Availability

The data presented in this study are available on request from the corresponding author. The data are not publicly available due to ongoing research by the authors.
